# Integrated Metabolomics and Network Pharmacology Reveal the Active Components and Potential Health-Beneficial Mechanisms of Different Jujube (*Ziziphus jujuba*) Cultivars

**DOI:** 10.3390/foods15081347

**Published:** 2026-04-13

**Authors:** Yuanru Chen, Xueling Zeng, Wei Qin, Wenjuan Geng, Ruocheng Tang, Ximo Bai, Weiquan Zhou

**Affiliations:** 1 College of Horticulture, Xinjiang Agricultural University/Xinjiang Institute of Jujube Industrial Development/Xinjiang Engineering Research Center for Efficient Cultivation and High-Value Utilization of Forest and Fruit Crops, Urumqi 830000, China; cyr702610@163.com (Y.C.); xuelingz0118@163.com (X.Z.); xjqinwei@163.com (W.Q.); gwj0526@163.com (W.G.); trc1819618@163.com (R.T.); b19801708736@163.com (X.B.); 2 College of Agriculture, Xinjiang Hetian College, Hotan 848000, China

**Keywords:** Chinese jujube fruit, widely targeted metabolomics, metabolite, pharmacological components, molecular docking

## Abstract

The health benefits of jujubes are closely related to their active substances. In this study, the active substances in ‘huizao’ (HZ), ‘junzao’ (JZ), ‘hamidazao’ (HMDZ), ‘kashigerexiaozao’ (KSGEXZ) and ‘yuanlingzao’ (YLZ) jujube varieties were identified via ultrahigh-performance liquid chromatography–tandem mass spectrometry (UPLC–MS/MS). After identification, a total of 1583 metabolites were found in jujube fruits, covering 13 major categories including alkaloids, amino acids and their derivatives. Pairwise comparisons revealed 518–755 differentially abundant metabolites across cultivars, and each variety had its main dominant metabolites. Through network pharmacology, 141 potential disease-related bioactive compounds were screened out, and key compounds were identified through molecular docking. These substances bind to the key target TP53 through hydrogen bonding and hydrophobic interactions. Among them, catechin, naringenin, and coniferin had the strongest binding affinities. These data increase our understanding of the active components of jujube and provide valuable information for the sustainable cultivation of jujubes for health applications.

## 1. Introduction

Jujube (*Ziziphus jujuba* Mill.) is a typical economic tree species native to China that has been utilized and cultivated for more than 7000 years; moreover, jujubes, peaches, plums, chestnuts and apricots are known as the five ancient fruits [1,2,3]. Jujube plants are characterized by their strong adaptability and high economic value, have been widely introduced into various regions, and occupy an important position in local agricultural production [4,5]. Notably, Xinjiang has developed into one of the largest jujube-growing regions in China, with hundreds of jujube germplasm resources, including the main varieties, such as ‘huizao’ (HZ) and ‘junzao’ (JZ), as well as decidedly local varieties, such as ‘hamidazao’ (HMDZ) and ‘kashigerexiaozao’ (KSGEXZ) [6,7]. Jujube fruits are rich in nutrients, owing to their potential health benefits. Jujube fruits have long played an important role in the Chinese diet. Corresponding to its prolonged cultivation history, jujube has been extensively documented in ancient texts for its dual use as both a food and a medicinal resource. It is recorded in classical works, such as Shennong Bencao Jingand Compendium of Materia Medica, for its health-promoting properties and has been traditionally used in herbal formulations and as a nourishing foodstuff in daily cuisine [8]. Moreover, these jujube varieties have shown strong development and utilization potential in the field of functional foods, warranting their further exploration and study.

Jujube is a traditional medicine and food homology plant, and its active substances significantly contribute to promoting human health [9]. Notably, polysaccharides and flavonoids are active substances that are widely considered to have considerable health-promoting effects [10,11]. These active substances exhibit nutritional properties that play important roles in the prevention and alleviation of various diseases [12,13]. Similarly, phenolic acids and terpenoids also have good nutritional properties [14,15], and related nutritional properties have also been reported in other fruits [16,17,18]. However, most recent research has focused on specific active substances, whereas comprehensive analyses remain limited. Because all the active components of jujube fruit are essential for its health benefits [19], it is necessary to conduct a comprehensive and in-depth metabolomic study on the composition of active substances in different jujube fruits to better understand their effects and mechanisms and provide a more solid theoretical basis for the further development and utilization of jujube fruits for nutritional applications.

Widely targeted metabolomics is a high-throughput and highly sensitive method for the comprehensive detection of metabolites [20]. It combines the high-throughput and universality of the nontargeted metabolomics approach with the high sensitivity and accuracy of targeted metabolomics [21]. Notably, accurate qualitative and quantitative analyses of substances can be achieved with a high-resolution triple quadrupole mass spectrometer in multiple reaction monitoring mode to explore the properties and functions of metabolites in depth [22]. Widely targeted metabolomics has been applied in crop genetic determinations [23], mechanistic research on plant pests and diseases [24] and food science applications [25]. At its core, network pharmacology integrates multidisciplinary approaches, including bioinformatics and computational pharmacology. This methodology employs high-throughput data mining to systematically decipher the synergistic mechanisms of multicomponent drugs. Specifically, active compounds were screened, and their targets were predicted using the TCMSP, SwissDrugDesign, and SymMap databases, while disease/symptom-related targets were retrieved from DisGeNET, SymMap, and TTD. Potential compound targets identified through this integrated platform were then intersected with disease targets. A component–target–pathway network was subsequently constructed via Cytoscape, (version 3.9.1, Cytoscape Consortium, San Diego, CA, USA) with KEGG pathway enrichment analysis revealing core signalling pathways. This holistic strategy establishes a novel research paradigm for elucidating the multicomponent, multitarget health-promoting properties of jujube fruits [26,27].

In this study, we employed the widely targeted metabolomics UPLC–MS/MS technique to conduct comprehensive qualitative and quantitative analyses of five different varieties of jujube fruits, comparing the differences in metabolite composition and relative content among jujube fruits. Principal component analysis (PCA) was used to visualize the data distribution, and potential active substances were screened through orthogonal partial least squares discriminant analysis (OPLS-DA) combined with variable importance in projection (VIP) values. Hierarchical cluster analysis (HCA) was utilized to reveal the coexpression characteristics of the metabolites and the clustering rules of the samples; moreover, the Kyoto Encyclopedia of Genes and Genomes (KEGG) database was integrated for enrichment analysis of metabolic pathways. To further explore the potential health-related value of jujube fruits, we combined network pharmacology and molecular docking methods to preliminarily screen bioactive components in jujube fruits that may be associated with health-related effects, with the aim of generating exploratory hypotheses for subsequent research and functional validation. This study helps further clarify the characteristic bioactive components of jujube fruits and provides preliminary theoretical support and referential insights for subsequent research on jujube fruits in nutritional food development and health-related functional exploration. The findings mainly provide hypotheses for further experimental validation and suggest potential biological relevance of the screened components to health-related properties.

## 2. Materials and Methods

### 2.1. Plant Samples and Quality Indicators

In this study, 5 jujube varieties, ‘kashigerexiaozao’ (KSGEXZ), ‘hamidazao’ (HMDZ), ‘huizao’ (HZ), ‘junzao’ (JZ) and ‘yuanlingzao’ (YLZ), were selected. Each cultivar had three biological replicates, sampled from three distinct healthy jujube trees, yielding a total of 15 biological samples across all five cultivars; each biological replicate extract was analyzed as described. All fruits were harvested from the Jujube germplasm resource nursery in Yuephu County, Kashgar region (39°14′ N, 76°52′ E). Considering the significant impact of different environmental conditions on the metabolic characteristics of jujube fruits, all jujube varieties were harvested uniformly after they matured in mid-October. The harvesting process was conducted within the same seasonal period and under consistent environmental conditions to ensure the stability of fruit quality in an orchard that uses conventional chemical fertilizer and water management practices. The harvested jujube fruit samples were divided into two parts. One part was used for component analysis. For this purpose, the flesh was separated by peeling and enucleating the fruits with a knife, after which the flesh was chopped, quickly frozen in liquid nitrogen, and stored at −80 °C. The other jujube fruit samples were used for appearance index determination. In this study, a digital Vernier calliper (Shanghai Shengguang Measuring Tool Co., Ltd., Shanghai, China) was used to measure the longitudinal and transverse diameters of the jujube fruits, and an electronic balance (MP2001, Shanghai Hengping, Shanghai, China) was used to measure the single-fruit weight of the jujube fruits.

### 2.2. Widely Targeted Metabolomic Analysis

#### 2.2.1. Sample Preparation and Extraction

After the samples were vacuum freeze-dried in a Scientz-100F freeze dryer, they were transferred to an MM400 grinder (Retsch Haan, Germany) and ground at 30 Hz for 1.5 min until a powdered form was obtained. Specifically, 50 mg of the powder was weighed, 200 μL of a precooled (−20 °C) 70% methanol aqueous solution containing internal standards was added for extraction, and the mixture was vortexed every 30 min; this process was repeated six times to ensure complete extraction. After the extraction was complete, the mixture was transferred to a centrifuge and centrifuged at a speed of 12,000 rpm for 3 min. The supernatant was collected and filtered through a microporous membrane with a pore size of 0.22 µm. The resulting filtrate was subsequently subjected to UPLC–MS/MS analysis.

#### 2.2.2. UPLC–MS/MS Analysis

In this study, an ExionLC™ AD (Sciex, Framingham, MA, USA) UPLC instrument equipped with an Agilent SB-C18 column (Agilent Technologies, Santa Clara, CA, USA; 2.1 mm × 100 mm, 1.8 μm particle size) was used for analysis of the sample extract. Mobile phase A was 0.1% formic acid in water, and mobile phase B was 0.1% formic acid in acetonitrile. The gradient programme was set as: 5% B at 0 min, linearly increased to 95% B over 9 min, held for 1 min, then decreased to 5% B from 10 to 11 min, and finally equilibrated at 5% B for 14 min. The flow rate was 0.35 mL/min, the column temperature was maintained at 40 °C, and the injection volume was 2 μL. Analysis was performed according to the methods of Li et al. [20].

Mass spectrometric detection was conducted using electrospray ionization (ESI) in both positive and negative modes. The ion source temperature was set at 550 °C, with ion spray voltages of 5500 V (positive) and −4500 V (negative). The nebulizing gas (GS1), heating gas (GS2), and curtain gas pressures were optimized at 50, 60, and 25 psi, respectively. Analysis was performed in multiple reaction monitoring (MRM) mode on a triple-quadrupole (QQQ) mass spectrometer, using nitrogen as the collision gas. The optimal MRM transitions, declustering potentials (DP), and collision energies (CE) for each target metabolite were established by direct infusion of authentic chemical standards and are documented in our self-built database. During sample analysis, specific MRM transitions were monitored within scheduled time windows based on the known retention times of metabolites to maximize sensitivity and specificity. The MS parameters were adapted from Wang et al. [25].

#### 2.2.3. Qualitative and Quantitative Metabolite Analyses

In this study, qualitative and quantitative analyses were conducted on the metabolites of jujube fruits based on the method of Zhang et al. [28].

Qualitative analysis: Metabolite identification was performed by matching experimental data with the self-built MWDB database (MetWare Database). This database was constructed using authentic chemical standards, and each entry contains reference data acquired under identical analytical conditions. All annotated metabolites were categorized into three confidence levels following standard MSI guidelines: (1) Level 1: metabolites fully confirmed with authentic reference standards by matching accurate mass, MS/MS fragments, retention time, MRM transitions, DP and CE parameters; (2) Level 2: high-confidence putative annotations based on rigorous MS/MS spectral and retention-time matching against the self-built database without available authentic standards; (3) Level 3: tentative putative classifications limited to compound family-level identification only. For a high-confidence annotation, a match was required for both high-confidence secondary mass spectrometry (MS/MS) and retention time (RT) data. During data processing, the interference signals generated by K^+^, Na^+^, and NH_4^+^_ admixture ions and fragment ions were excluded from false-positive annotations. Detailed confidence classification for each identified metabolite is listed in Table S1.

Quantitative analysis: Relative quantification of the identified metabolites was achieved by integrating the peak areas of their corresponding MRM transitions. The results are expressed as relative content. To ensure data quality and reproducibility, a rigorous quality control (QC) protocol was implemented. A pooled QC sample (a mixture of equal aliquots from all experimental samples) was analyzed at regular intervals throughout the analytical sequence. This enabled monitoring of instrument stability, RT alignment, and correction for minor signal intensity drift. The final relative abundance for each metabolite was calculated from the corrected chromatographic peak area and used for subsequent statistical and comparative analyses.

### 2.3. Identification of the Key Active Components of Jujube Fruits and Prediction of Their Disease Targets

This study utilized three databases, namely TCMSP, SwissDrugDesign, and SymMap, to screen the key active components in jujube fruits and predict their targets. On the TCMSP platform (https://tcmspw.com/tcmsp.php, accessed on 1 July 2024), key active substances were screened based on the criteria of oral availability (OB) ≥ 30% and drug similarity (DL) ≥ 0.18. Further screening was performed through the SwissDrugDesign SWISSADME (http://www.swissadme.ch/, accessed on 1 July 2024) module, and the targets were predicted with its SwissTargetPrediction module (http://www.swisstargetprediction.ch/, accessed on 1 July 2024). We also used the SymMap database (http://www.symmap.org/, accessed on 1 July 2024) to collect information on disease targets. Through cross-validation and comprehensive analysis of multiple databases, comprehensive information on the active components and their targets in jujube fruits was mined to the greatest extent, providing rich and reliable data support for in-depth exploration of the pharmacological mechanism of jujube fruits.

### 2.4. Molecular Docking

For the molecular docking analysis, the 3D structures of the three ligand compounds were obtained from the PubChem database (https://pubchem.ncbi.nlm.nih.gov/) and converted to PDB format using OpenBabel (version 3.1.1, OpenBabel Development Team). Ligands were further processed by adding Gasteiger charges, merging non-polar hydrogens, and setting rotatable bonds for flexible docking. The crystal structure of the target protein TP53 (PDB ID: 3D06) was downloaded from the RCSB PDB database (https://www.rcsb.org/). This structure was selected for docking due to its high resolution and well-characterized conformation of the TP53 DNA-binding domain, which is the primary functional region for ligand interaction. Receptor preparation was performed using PyMOL (version 2.5.0, Schrödinger, LLC, New York, NY, USA) to remove water molecules, ions, and non-protein heteroatoms. Polar hydrogens and Gasteiger charges were added to the protein using AutoDockTools (version 1.2.3, The Scripps Research Institute, La Jolla, CA, USA). Since no co-crystallized ligand was present in the 3D06 structure to define a specific binding pocket, the docking search space (grid box) was defined to cover the entire surface of the TP53 protein for global blind docking, ensuring comprehensive screening of potential binding sites. Docking was performed using AutoDock Vina (version 1.2.3, The Scripps Research Institute, La Jolla, CA, USA)with default parameters (exhaustiveness = 10). The top 10 conformations of each ligand were output, and the conformation with the lowest binding energy (kJ·mol^−1^) was selected as the optimal result. The binding modes, including hydrogen bonds, hydrophobic interactions, and spatial matching, were visualized using PyMOL (version 2.5.0, Schrödinger, LLC, New York, NY, USA) and LigPlot (version 2.2, European Bioinformatics Institute, Hinxton, UK). This molecular docking method was adapted from Peng et al. [29].

### 2.5. Data Analysis

Statistical analysis was conducted using Microsoft Excel 2019 (Microsoft Corporation, Redmond, WA, USA), IBM SPSS 27 (IBM Corp., Armonk, NY, USA) and R software (version 4.3.1, R Foundation for Statistical Computing, Vienna, Austria). Prior to all analyses, technical duplicate data were averaged for each biological sample, and only three independent biological replicates per cultivar were used for statistical evaluation. Statistical analysis was performed on all three sets of biological replicate data. PCA was conducted using the built-in statistical prcomp function of R software. HCA was conducted using the ComplexHeatmap package of R software. For OPLS-DA, modelling was performed following the standard workflow of Metware metabolomics platform (Metware Biotechnology Co., Ltd., Wuhan, China); data were preprocessed with logarithmic transformation and UV scaling, The optimal predictive and orthogonal components were automatically determined. The *p*-values for pairwise comparisons were calculated using Student’s *t*-test, and the Benjamini–Hochberg false discovery rate correction was applied for multiple testing. The KEGG database (https://www.kegg.jp/kegg/, accessed on 1 July 2024) was used for functional annotation of differential metabolites and enrichment analysis of metabolic pathways rather than determining metabolite concentrations. Differentially abundant metabolites were screened according to the criteria: VIP ≥ 1.0, fold change (FC) ≥ 2 or ≤0.5, and corrected *p* value ≤ 0.05.

## 3. Results

### 3.1. Analysis of the Appearance and Quality of Five Varieties of Jujube Fruits

**Figure 1:**
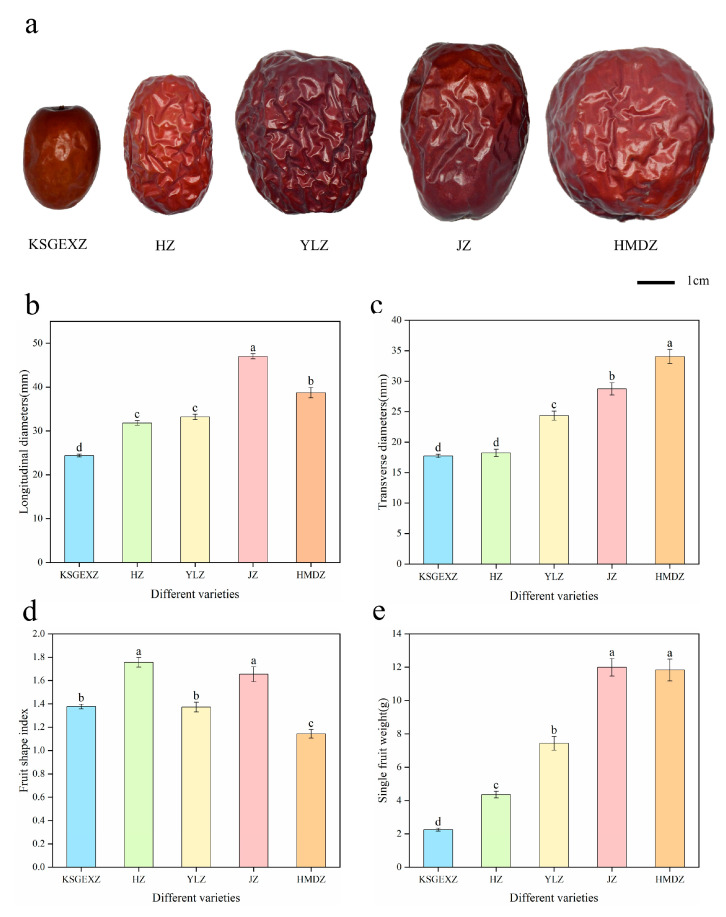
Comparison of fruit quality indices of the five jujube varieties. (**a**) Morphological characteristics of the five jujube varieties. (**b**) Longitudinal diameters of the fruits. (**c**) Transverse diameters of the fruits. (**d**) Fruit shape indices. (**e**) Single-fruit weights. Different lowercase letters in the figure represent significant (*p* < 0.05) differences between varieties.

As shown in Figure 1, the phenotypic characteristics of different jujube varieties at maturity significantly differed. Specifically, the longitudinal diameter of JZ is significantly greater than that of the other varieties. The HMDZ is characterized by the maximum transverse diameter and single-fruit weight. The fruit shape index further revealed the phenotypic patterns of the varieties. HZ has a typical long cylindrical feature, whereas the fruit shape index of KSGEXZ is 1.378, and that of YLZ approaches 1.374, indicating that both exhibit similar oval-shaped fruit shapes. In terms of the single-fruit weight indices, the single-fruit weights of JZ and HMDZ were significantly greater than those of the other varieties, and the range of single-fruit weights of these varieties was between 2.3 g and 12 g. The abovementioned indicators reflect the differences in fruit shape among different varieties of jujube fruits. For example, the fruit of the Kashgar jujube was small and oval-shaped, whereas the fruit of the Hami jujube was relatively large and nearly round. In summary, significant differences were observed in the external quality traits among the different jujube fruit varieties evaluated in this study.

### 3.2. Analysis of the Metabolites in the Five Varieties of Jujube Fruits

Based on the close association between the health benefits of jujube fruits and their metabolites, in this study, extensive targeted UPLC–MS/MS metabolomics technology was adopted to analyze the metabolic differences among the five main cultivated jujube varieties systematically. Whole-process quality control was carried out by inserting QC samples, and the overlap rate of the positive and negative ion current diagrams (TICs) was relatively high, verifying the stability of the instrument (Figure S1). TIC overlaps and low coefficient of variation (CV < 0.5 for 85% of the metabolites) confirmed the reliability and reproducibility of the method (Figure S2).

**Figure 2:**
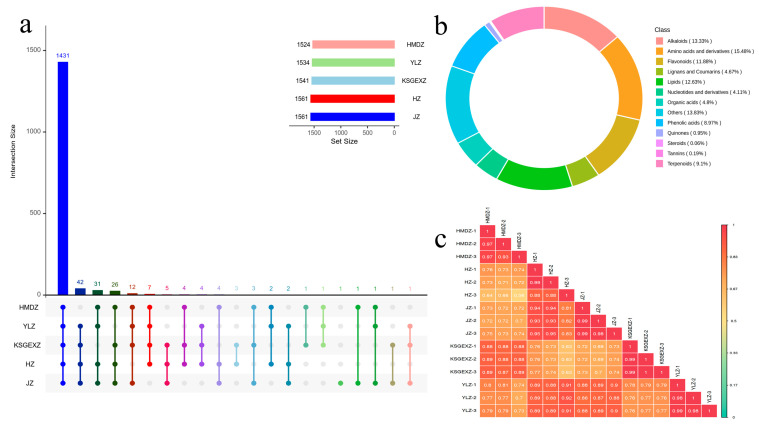
Metabolomics analysis of the fruits of five jujube cultivars. (**a**) Upset diagram of the number of metabolites. (**b**) Ring diagram depicting the metabolite categories. (**c**) Metabolite correlation analysis.

This study analyzed and revealed the metabolite components of five jujube varieties. Among the 1583 metabolites detected, 1431 were common metabolites across all varieties. In the specific analysis of each variety, HZ and JZ ranked first, with 1561 metabolites, which included 37 more metabolites than HMDZ (1524). Quercetin-3-O-rhamnoside and N-methyl-L-glutamic acid are unique to HZ, JZ, and YLZ. Second, the KSGEXZ variety contains 1541 metabolites, including quercetin-3-O-xylityl (1 → 2) glucosyl (1 → 2) glucoside, which is shared with HMDZ (Figure 2a). These detected metabolites can be classified into 13 categories, among which amino acids and their derivatives (15.48%), other categories (13.83%), and alkaloids (13.33%) constitute the core metabolic components. Notably, lipids (12.63%), flavonoids (11.88%), terpenoids (9.1%), phenolic acids (8.97%), organic acids (4.80%), lignans and coumarins (4.67%), nucleotides and derivatives (4.11%), quinones (0.95%), tannins (0.19%), and steroids (0.06%) were also significantly enriched. The combined proportion of the remaining categories (organic acids, lignans and coumarins, nucleotides and their derivatives) was less than 15%, and their distribution characteristics may reflect the regulatory differences in various specific metabolic pathways (Figure 2b, Table S1). Although the above 13 types of metabolites can be detected in jujube fruits, there were significant differences in the relative contents of active substances in different jujube fruits. Through correlation analysis of different metabolites of jujube fruits (Figure 2c), the results revealed that the HZ and JZ varieties presented relatively similar characteristics (0.81 ≤ |r| ≤ 0.95). The low correlation values between HZ and HMDZ ranged from 0.56 to 0.76, which may stem from their different origins and variety backgrounds.

### 3.3. Multivariate Statistical Analysis of Fruit Metabolites of Five Jujube Cultivars

**Figure 3:**
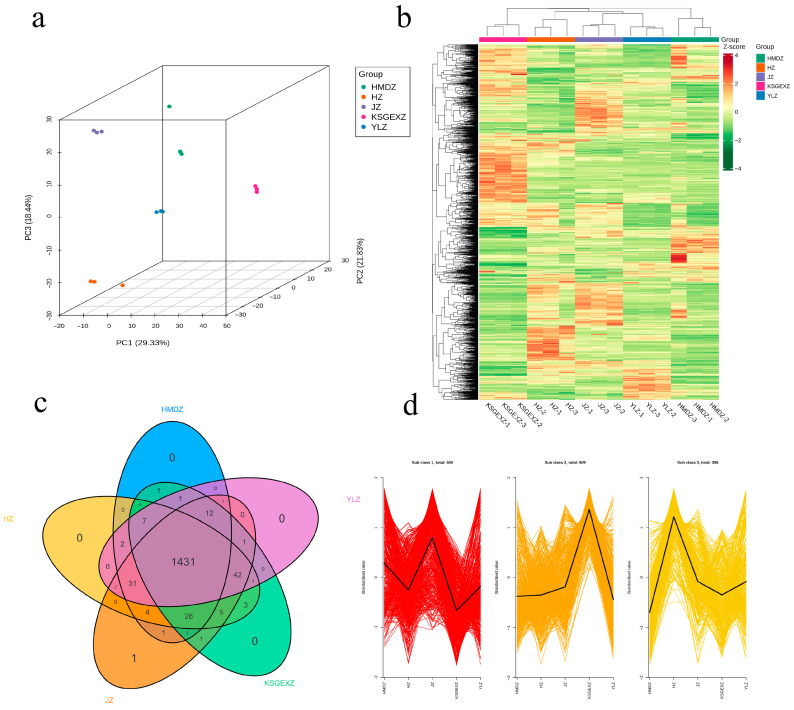
Multivariate statistical analysis of fruit metabolites of five jujube cultivars. (**a**) Principal component analysis PCA diagram; (**b**) Cluster analysis of HCA diagram; (**c**) Venn diagram of metabolites; (**d**) K-Means analysis of differential metabolites.

To analyze the metabolite differences in fruits of different jujube varieties, in this study, the metabolomic data of the five varieties were evaluated through unsupervised identification PCA (Figure 3a). The results revealed that the cumulative contribution rate of the first two principal components was 51.16% (PC1 = 29.33%, PC2 = 21.83%), reflecting the primary metabolic variation among the jujube varieties. The PCA plot shows that KSGEXZ was significantly separated from the other four varieties on the PC1 axis, whereas the PC2 axis further distinguished HMDZ from HZ. There was a clear separation trend between the varieties, clearly reflecting that there were certain differences in the relative abundance and accumulation levels of metabolites among different jujube fruits. HCA based on the relative contents of metabolites revealed that all the samples could be significantly divided into six subgroups (Figure 3b). The KSGEXZ variety was active in the (cluster I) and (cluster III) synthetic pathways, whereas the highest levels were expressed in HMDZ (cluster IV) and YLZ (cluster VI), and the content of cluster V peaked in the HZ variety. Based on the results of the PCA and HCA plots, there were significant differences in metabolites between the different groups. However, the Venn analysis results revealed that the metabolite compositions of the fruits of the five varieties were highly similar (Figure 3c).

### 3.4. Analysis of Different Metabolites in the Jujube Fruits of the Five Cultivars

Pairwise OPLS-DA was applied to compare metabolite profiles among five jujube cultivars for identifying differential metabolites. All models presented high performance (R2Y = 1, Q2 = 0.982–0.993). The latent component number was standardized in model construction; permutation verification and cross-validation were conducted to evaluate overfitting. The attached validation plots reflect the permutation test outcomes, proving that the high R2Y and Q2 values are supported by reliable model validation. Combined with the significant separation phenomenon shown by the grouped PCA pairings (Figures S3 and S4), these findings intuitively indicate that there were significant differences in metabolites between various varieties. The differentially abundant metabolites were screened according to the criteria of variable importance in the projection (VIP) ≥ 1 and fold change (FC) ≥ 2 or FC ≤ 0.5, and the screening results were presented through volcano maps. The specific data are as follows: In the pairing comparison between the KSGEXZ and HMDZ/HZ/JZ/YLZ varieties, 725–755 significantly different metabolites were detected. In the paired comparison between the YLZ and HMDZ/HZ/JZ variety groups, 536, 558, and 580 significantly different metabolites were identified, respectively. When the combination pairs of HMDZ and HZ/JZ varieties were compared, 629 and 634 significantly different metabolites emerged, respectively. However, the comparison between the JZ and HZ variety groups revealed 518 differentially abundant metabolites (Figure S5, Table S2). These data fully reflect that there are significant differences among different jujube varieties in terms of the composition of metabolites and their regulation during the same period.

In this study, the K-means analysis method was adopted to conduct cluster analysis on the relative contents of 1365 different metabolites. The results revealed that these metabolites could be divided into three subcategories. Significant differences in the content distribution of differentially abundant metabolites were observed among the jujube varieties: 500 metabolites in the JZ variety and 509 metabolites in the KSGEXZ variety showed relatively high contents, while 356 metabolites in the HZ variety were characterized as differentially abundant. Overall, the KSGEXZ variety exhibited the highest content of differentially abundant metabolites (Figure 3d). These results provide an important basis for elucidating the unique metabolic characteristics and chemical diversity of the KSGEXZ jujube variety.

#### 3.4.1. Alkaloids

**Figure 4:**
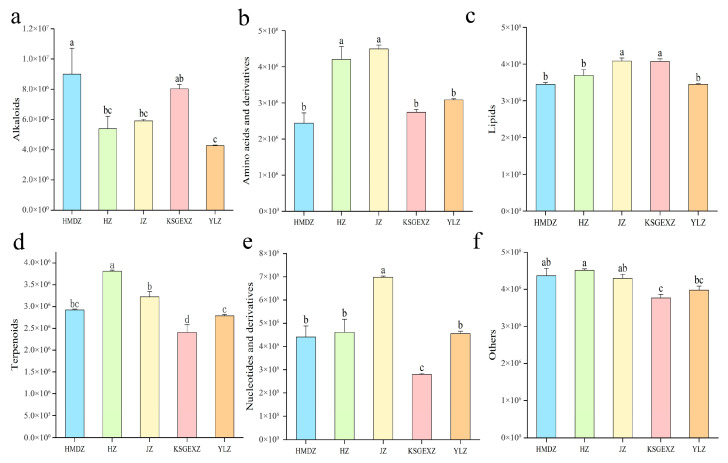
Histogram of the main active substances in the fruits of the five jujube varieties. (**a**) Relative contents of alkaloids. (**b**) Relative contents of amino acids and their derivatives. (**c**) Relative contents of lipids. (**d**) Relative contents of terpenoids. (**e**) Relative contents of nucleotides and their derivatives. (**f**) Relative contents of other categories of metabolites. Different lowercase letters above the bars indicate significant differences at *p* < 0.05 according to Tukey’s multiple range test.

In this study, a total of 12 types of alkaloid metabolites, including isoquinoline, indole, apolphine, and pyridine alkaloids, were detected in the 5 jujube varieties. A systematic comparison revealed that there were significant differences among the different varieties, and they had patterned characteristics. When KSGEXZ was compared with HMDZ/HZ/JZ in pairs, the number of differential alkaloids (69–109 kinds) continuously increased, and the number of upregulated alkaloids (49, 58, 62) was greater than the number of downregulated alkaloids. However, YLZ exhibited the opposite regulatory pattern. Compared with those of the four varieties in pairs, the number of alkaloids (69–122) differed. In contrast to the KSGEXZ variety, the number of alkaloids regulated by the YLZ variety exceeded those regulated by the other varieties. Compared with the HZ/HMDZ varieties, 46 and 97 differentially abundant metabolites were identified in JZ, respectively. Compared with the HMDZ variety, 89 differentially abundant metabolites were identified in the HZ variety (Table S3). During the entire analysis process, isoquinoline alkaloids were a focus of the HMDZ variety. HZ is dominated by indole alkaloids, including substances such as betacyanin and ergotamine (Table S1). This study also compared the relative contents of different varieties of alkaloids. The results revealed that the relative contents of alkaloids in the HMDZ and KSGEXZ varieties were significantly greater than those in the other varieties (Figure 4a). These findings not only reveal the differences in alkaloids among the different jujube varieties but also provide a basis for further research on the bioactivities and potential applications of jujube fruits.

#### 3.4.2. Amino Acids and Their Derivatives

Amino acids and their derivatives, the basic units of proteins, are important nutritional components of jujube fruits, as they maintain the nutritional balance of jujube fruit. Thus, a comprehensive comparative analysis was performed to explore the differences in nutrition among the different jujube varieties. This study systematically analyzed the differences in the metabolites of amino acids and their derivatives among the five jujube varieties. In the pairwise comparison between the KSGEXZ and HMDZ/HZ/JZ varieties, 100, 120, and 139 differentially abundant metabolites were detected, respectively. When the YLZ variety was paired and compared with the HMDZ/HZ/JZKSGEXZ varieties, the results were equally rich, with 84, 83, 117 and 108 differentially abundant metabolites, respectively. However, when the HZ variety was compared with the HMDZ variety, 91 genes were identified, 73 of which were upregulated and 18 of which were downregulated (Table S3). Moreover, the relative content of L-proline in HMDZ was significantly greater than that in the other varieties. In JZ, the contents of L-lysine, L-tyrosine and L-phenylalanine were relatively high; these amino acids are crucial for improving the quality of jujube fruit protein (Table S1). Compared with the other varieties, the JZ and HZ varieties presented significantly greater levels of amino acids and their derivatives (Figure 4b). These findings not only help reveal the unique biological characteristics of different jujube varieties but also lay a theoretical foundation for the development of jujube-based functional foods.

#### 3.4.3. Lipids

A systematic analysis of six types of lipid metabolites, including free fatty acids, lysophosphatidylethanolamine, glycerides, phosphatidylcholine, sphingolipids, and lysophosphatidylcholine, detected in jujube fruits was conducted. Among them, free fatty acids were the most abundant. The differential lipid metabolites of the various varieties were also studied and compared. In the paired comparison of the KSGEXZ and HMDZ/HZ/JZ varieties, the number of lipid metabolites differed (107, 65, and 63 kinds), among which lysophosphatidylcholine and lysophosphatidylethanolamine were significantly enriched in KSGEXZ. When paired and compared with the YLZ and HMDZ/HZ/JZ/KSGEXZ varieties, 74, 69, 77, and 92 differences in lipid metabolites were identified. For the pairing analysis of the JZ and HZ/HMDZ varieties, the number of lipid metabolites differed (64 and 90, respectively). When the HZ and HMDZ varieties were compared, 85 differential lipid metabolites were identified (Tables S1 and S3). This study also conducted a comprehensive comparative analysis of the relative lipid contents in the fruits of the five jujube varieties. The results revealed that the JZ variety ranked first in terms of the relative lipid content, and free fatty acids were significantly enriched, indicating that its energy metabolism was relatively active (Figure 4c). These results not only provide key data for a deeper understanding of the lipid composition of jujube fruits and the differences among varieties but also lay a solid foundation for the application and development of jujube fruits in the fields of food and nutrition.

#### 3.4.4. Terpenoids and Nucleotides and Their Derivatives

This study analyzed terpene compounds in the five jujube varieties and identified six types of terpene substances, including monoterpenoids, sesquiterpenoids, diterpenoids, triterpenoids, triterpenoid saponins, and terpenes. Among them, the content of triterpenoid metabolites was the highest. The content of terpenoids in HZ was significantly greater than that in the JZ, HMDZ, YLZ and KSGEXZ varieties, in sequence (Figure 4d). The HZ variety exhibited unique advantages in terms of the accumulation of triterpenoids. The KSGEXZ variety had an advantage in terms of the relative contents of diterpenoids and sesquiterpenoids. In addition, the relative contents of nucleotides and their derivatives in the JZ variety were significantly greater than those in the other varieties. Among them, the enrichment levels of guanosine and adenine were particularly prominent (Figure 4e, Table S1). These research results provide crucial evidence for a deeper understanding of the metabolite differences among jujube varieties. These findings not only help reveal the unique biological characteristics of different jujube varieties but also offer important theoretical support for the further development and utilization of jujube fruits in fields such as food and medicine.

#### 3.4.5. Other Compounds

The material components of the fruits of the five jujube varieties were also analyzed. Using professional detection methods, nine other types of substances, including saccharides, alcohol compounds, ketone compounds, aldehyde compounds, vitamins, stilbenes, chromones, lactones, and other unclassified components. Paired analysis revealed that there were significant differences between the different varieties. In the pairing comparison between the KSGEXZ and HMDZ/HZ/JZ varieties, the quantity and changing trends of differentially abundant metabolites were clearly presented (105, 90, 86 types), and overall, there was a trend that the number of downregulated metabolites was greater than the number of upregulated metabolites. When YLZ was paired and compared with the HMDZ/HZ/JZ/KSGEXZ varieties, the 66, 55, 64, and 88 differentially abundant metabolites were identified. In the comparison between the JZ and the HZ/HMDZ varieties, 55 other types of differentially abundant metabolites were identified, but there were significant differences in the proportions of upregulated and downregulated metabolites. When the HZ and HMDZ varieties were compared, 68 other types of differentially abundant metabolites were identified (Table S3). Further analysis revealed that the relative contents of other types of substances in the HZ were significantly greater than those in the other varieties (Figure 4f). Sugars are an important type of active substance in jujube fruits, and analysis revealed that there were significant differences in the relative sugar contents. The relative sugar contents of HZ and HMDZ were relatively high. The content of inositol galactoside in JZ was particularly prominent. The content of L-glucose * in KSGEXZ was relatively high. The differences in sugars among jujube varieties not only affect fruit flavour but are also directly related to their functional characteristics. The different varieties also significantly differed in terms of ketone and alcohol contents. The relative vitamin contents of the HMDZ and JZ varieties were greater than those of the other varieties (Table S1).

### 3.5. KEGG Annotation and Enrichment Analysis of the Differentially Abundant Metabolites

In this study, through KEGG pathway enrichment analysis, the top 20 significant metabolic pathways of differentially abundant metabolites among the five jujube varieties were identified (*p* value < 0.05), and bubble charts were constructed (Figure S6). In the paired comparisons of different varieties, the number of differential metabolic pathways involved was as follows: 76–86 for KSGEXZ compared with other varieties, 73–81 for YLZ compared with other varieties, 80–84 for HMDZ compared with HZ/JZ, and 73 for JZ compared with HZ.

**Figure 5:**
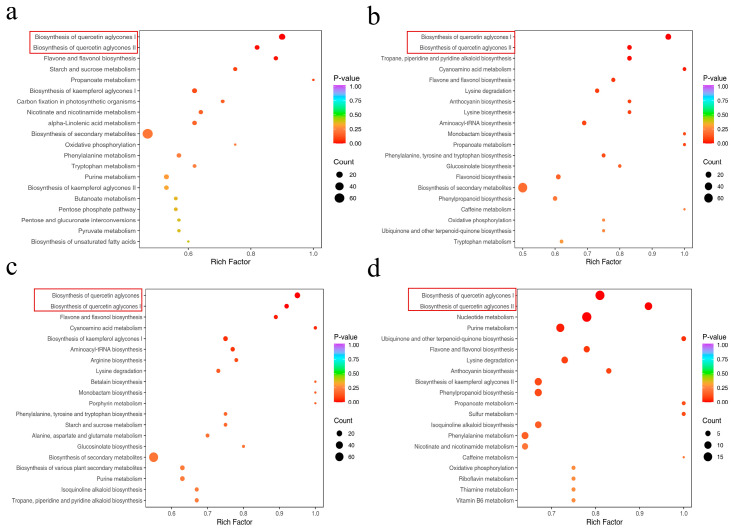
Pairwise comparisons of the differentially abundant metabolites in KSGEXZ fruits determined by KEGG analysis. (**a**) KSGEXZ vs. HMDZ. (**b**) KSGEXZ vs. HZ. (**c**) KSGEXZ vs. JZ. (**d**) KSGEXZ vs. YLZ.

This study revealed the key differences in the metabolic regulatory network among different jujube varieties through KEGG pathway enrichment analysis. Among them, the biosynthesis pathway of flavonoids (quercetin, glycoprotein I/II, flavonoid and flavonol metabolism) was specifically enriched in the KSGEXZ variety, which was highly consistent with its significantly increased relative content of flavonoids (Figure 5). At the amino acid metabolism level, the enrichment of the phenylalanine/tyrosine/tryptophan biosynthesis pathway in the HMDZ variety was directly related to its high amino acid content. In addition, the specific enrichment of the JZ variety in the purine metabolism pathway may affect the fruit development process by regulating the efficiency of protein synthesis and the level of energy metabolism. The differential regulation of these metabolic pathways jointly shapes the unique physiological characteristics of different jujube varieties. Among them, the enriched flavonols in KSGEXZ may be associated with favourable health-related properties and provide preliminary predictive clues for further exploratory functional validation, whereas the amino acid metabolic characteristics of HMDZ point to its application potential in the field of nutritionally fortified foods. These results not only reveal the metabolic differences among different jujube varieties but also provide clues for further study of the biological characteristics and quality mechanisms of jujube fruits.

### 3.6. Network Pharmacology Analysis

#### 3.6.1. Screening the Active Components and Substance Targets

**Figure 6:**
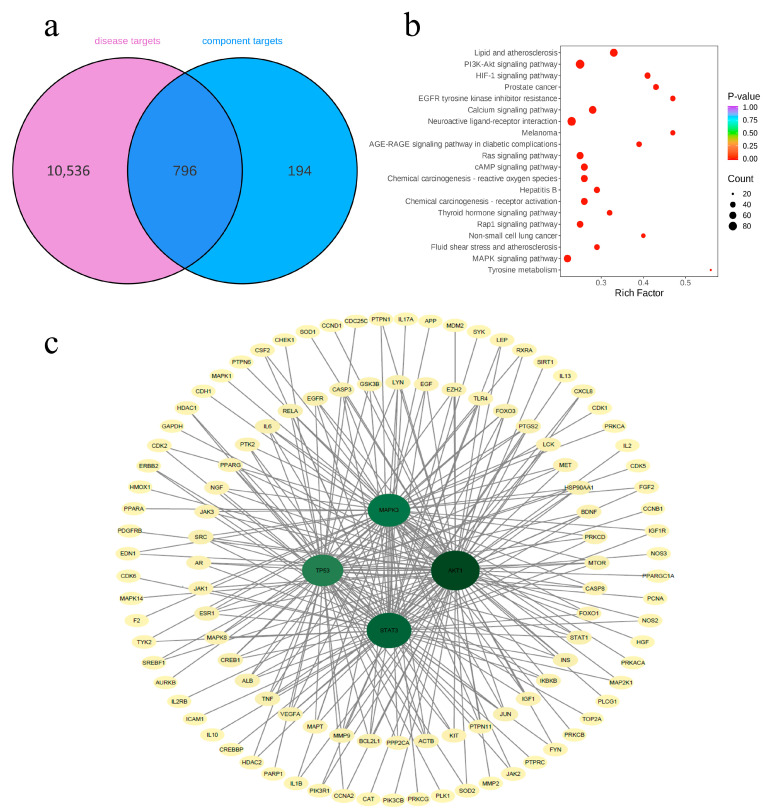
Screening the active components in the fruits of five jujube varieties. (**a**) Venn diagram of the substance targets and disease targets. (**b**) Maps of 20 target pathways with the most significant enrichment. (**c**) The “protein–protein interaction” network of the first 150 cross-targets.

In this study, network pharmacological calculations were used to predict potential interactions between the active components of the five jujube varieties and targets related to cancer and metabolic regulation. Owing to the complex composition of jujube fruits, determining which components play a key role in physiological functions is difficult. Based on the combined screening of the TCMSP, SwissDrugDesign and SymMap databases (DL ≥ 0.18, OB ≥ 30%), 194 potential targets related to the active substances of jujube fruits were identified from 1583 active substances (Figure 6a). These results provide important data for in-depth research on the mechanisms of action of active components in jujube fruits and their development in the fields of food and medicine.

#### 3.6.2. Screening Diseases and Disease Targets

Using the SymMap database and relevant literature, this study identified 10,536 disease-related targets associated with 46 diseases for which jujube fruits have potential application (Figure 6a, Table S4). To enhance the specificity of the analysis, a stringent screening was applied to both the compound targets and disease targets, with the component target parameter set at 0.12. A subsequent Venn diagram analysis revealed 796 overlapping targets between the compound and disease sets (Figure 6a). These intersection targets were linked to 141 active ingredients, which comprised 36 phenolic acids, 25 alkaloids, 27 compounds classified as others, 19 terpenoids, 16 flavonoids, and 12 lignans and coumarins.

To screen the core targets, a protein–protein interaction (PPI) network was constructed for the targets of the 796 active substances from the five cultivars using the STRING database combined with Cytoscape software, with TP53 identified as one of the key targets (Figure 6c). The PPI network consisted of multiple nodes, indicating that the key metabolites in jujube fruits may exert their biological functions through multiple regulatory targets and pathways. Topological analysis was performed within Cytoscape to evaluate node connectivity and importance. The average degree between nodes was 95.1, and the average eigenvector centrality was 0.144. The topological parameters of TP53 were higher than both average values mentioned above (Table S6), supporting its critical role as a core target in the network.

Furthermore, computational screening revealed associations between the disease targets and 312 signalling pathways. The figure shows the top 20 signalling pathways among them (Figure 6b). Notably, the disease-related signalling pathways were enriched mainly in the PI3K–Akt signalling pathway; the neuroactive ligand–receptor interaction; the neurodegenerative pathways, which include multiple disease targets; the lipid and atherosclerotic pathways; the calcium signalling pathways; and the MAPK signalling pathway. Moreover, the MAPK signalling pathway, as one of the main signalling pathways, is strongly correlated with TP53. The active substances in jujube fruit, identified through this study, provide a theoretical foundation and indicate promising directions for future research into their health-related applications.

#### 3.6.3. Molecular Docking of Key Active Substances with Core Targets

**Figure 7:**
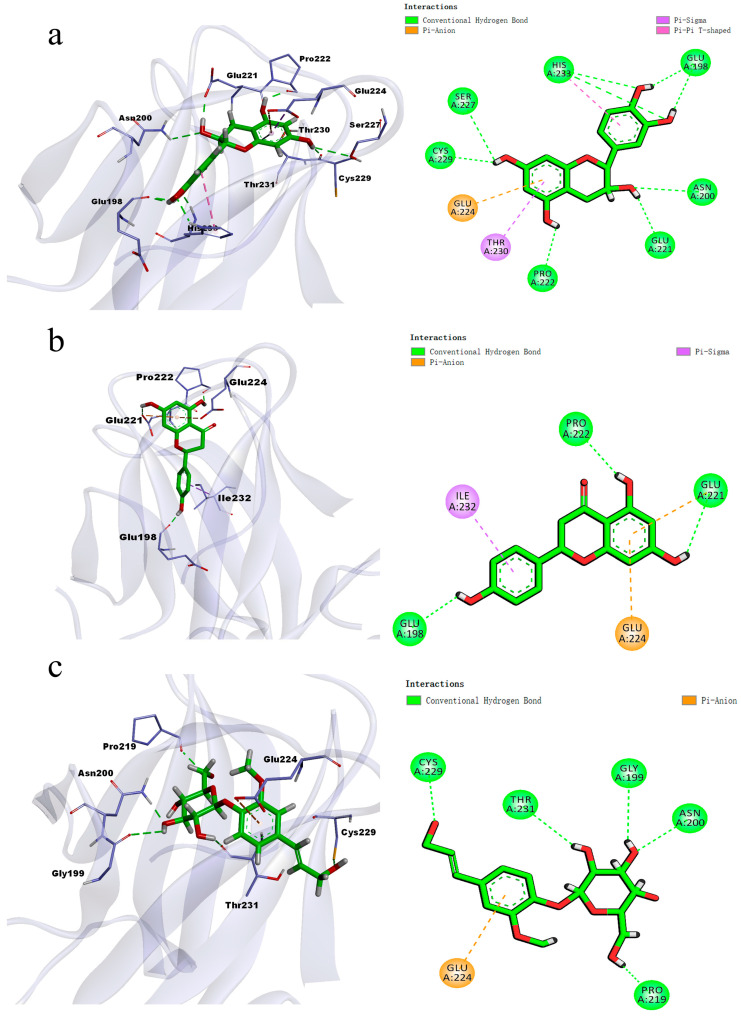
Molecular docking results of different metabolites. (**a**) Prediction of the binding mode of Catechin and TP53. (**b**) Prediction of the binding mode of Naringenin and TP53. (**c**) The binding mode of Coniferin with TP53.

The binding energies between eight key bioactive compounds in jujube fruits and the core key target TP53 were calculated using AutoDock Vina, with the exhaustive search parameter set to 10 to obtain optimal binding conformations. The results revealed that the binding energies of the flavonoids catechin and naringenin, as well as coniferin, with TP53 were −8.2, −6.8, and −6.6 kcal mol^−1^, respectively, ranking as the top three (Table S7). Catechins bind to TP53 through hydrogen bonds and hydrophobic interactions with residues such as GLU198, ASN200, and GLU221 (Figure 7a). Naringenin forms hydrogen bonds with GLU224 and ILE232 and forms hydrophobic interactions with residues such as GLU198 and GLU221 (Figure 7b). Coniferin forms hydrogen bonds with residues such as GLY199, ASN200, and PRO219 and forms hydrophobic interactions with GLU224 (Figure 7c). These results indicate that these active substances, especially catechins, have good binding affinity for TP53. The hydrogen bonding and hydrophobic interactions observed in these molecular docking analyses suggest hypothetical biological relevance to TP53-associated pathways, providing preliminary computational clues for further experimental validation. This study initially revealed the potential pharmacological value of the active components in jujube fruits, providing key clues and a theoretical basis for subsequent in vitro/in vivo experiments.

## 4. Discussion

Jujube, a traditional Chinese medicinal material that is both edible and medicinal, is rich in various active components, such as terpenoids and flavonoids, which are believed to contribute to its health-promoting properties. In recent years, with the increasing interest in the research of plant active substances, these components in jujube fruits have attracted much attention because of their multiple benefits in reducing the risk of various diseases and promoting human health [30]. However, at present, research aimed at pinpointing the components responsible for the specific health benefits of jujube fruits is relatively scarce. This scarcity hinders the precise characterization of its functional properties and the efficient utilization of its germplasm resources. Metabolomic technology, with its advantages of high-throughput and multivariate data analysis, can be used to analyze the composition of metabolites in organisms comprehensively. Its characteristics of high-throughput detection, wide coverage, and high-precision analysis make it an efficient means to explore the potential active components of medicinal plants [21,31]. Network pharmacology predicts the potential molecular association mechanism between active substances and therapeutic targets by constructing a “component–target–pathway” network. This method has been widely applied in the field of traditional Chinese medicinal materials research [25,32]. Molecular docking is a computer simulation method based on induced fit. By predicting the three-dimensional spatial binding modes and binding affinities of ligands and receptors, the intermolecular interaction mechanism can be revealed. This approach is widely adopted for the virtual screening of bioactive candidate compounds in natural product research. When combined with network pharmacology, it can help predict potential target interactions and underlying action mechanisms of ingredients that may be associated with health-related effects, aiming to provide hypotheses for further experimental validation and suggest potential biological relevance [29].

The combination of metabolomics and network pharmacology is an effective way to explore the active components of traditional medicinal plants effectively. For example, Sha et al. [33] reported that the leaves and flowers of Chinese pistache are rich in both nonvolatile and volatile metabolites with several potential health benefits, such as anti-inflammatory and antioxidant effects and the ability to protect the central nervous system. Zeng et al. [32] studied the differences in metabolites and the potential pharmacological mechanisms of safflower, which is used as both a medicine and food, from different regions and reported that safflower promotes blood circulation, removes blood stasis, and prevents cardiovascular disease and Alzheimer’s disease. Xia et al. [27] conducted a systematic study on the metabolomic characteristics and health-promoting effects of mature Camellia seeds from different geographical regions and identified 341 TCM chemical components from 1057 metabolites, among which 76 nonvolatile metabolites were identified as key active substances. Based on the above research results, in this study, UPLC–tandem mass spectrometry (UPLC–MS/MS) was used to conduct systematic qualitative and quantitative analyses of the active substances in the fruits of five jujube varieties, namely, HZ, JZ, KSGEXZ, YLZ, and HMDZ. A total of 1583 metabolites were identified in the present study, which could be classified into 13 major categories. Among them, alkaloids, amino acids, lipids, and terpenoids were determined to be the main active substances in jujube fruits. This study further highlights the crucial role of metabolomics in analyzing the functional components of plants that are both medicinal and edible.

The alkaloids in jujube fruits (such as quinolines, indoles, aporphine, and pyridine) are the key flavour substances that confer bitter taste characteristics. Metabolomic analysis has indicated that quinoline alkaloids contribute most significantly to bitterness [34]. In this study, a total of 211 alkaloids in 12 categories were identified in the 5 jujube varieties. Furthermore, 245 kinds of amino acids and their derivatives were also detected in this study. Among them, the contents of amino acids and their derivatives were relatively high in the JZ and HZ varieties. These bioactive substances provide a scientific basis for the targeted development of functional foods and drugs; moreover, studies have shown that amino acids and their derivatives are not only important aroma precursors in food but also key components that determine the flavour quality of food [35,36]. This further highlights the potential of developing jujube fruit as a multifunctional raw material. Notably, jujube fruits have been proven to be a potential source of natural terpenes for use in the development of functional foods, and significant differences in terpene composition were noted among the different jujube varieties [37]. In this study, 144 terpenes in 6 classes, such as monoterpenes, sesquiterpenes, diterpenes, triterpenes, and saponins, were identified in the 5 varieties of jujube fruits. Sarah et al. reported that terpenoids are the main active components in jujube fruits and have a wide range of medicinal applications [9]. The relative content of terpenoids in the HZ variety was significantly greater than in the other varieties. This chemical distinctiveness highlights HZ as a potential source of bioactive terpenoids for the development of functional products.

In this study, network pharmacology and molecular docking techniques were employed to predict and screen for components in the five jujube varieties that may be associated with health benefits. We screened a total of 141 metabolites with potential bioactivity and, through computational models, predicted that they might be associated with the regulation of cell proliferation and viability, metabolic processes, and cellular stress responses. The key metabolic substances were identified, and their interactions with the TP53 target were verified. Phenolic acids, alkaloids, and terpenoids were the most abundant compounds potentially contributing to the health-promoting properties of jujube fruits. The HZ variety was rich in phenolic acids and terpenoids. Among them, pentacyclic triterpenoids such as oleanolic acid and ursolic acid can exert anticancer effects by inducing the apoptosis of cancer cells and inhibiting angiogenesis [38]. The KSGEXZ variety has advantages in terms of the contents of flavonoids, lignans, and coumarins. The contents of phenolic acid substances in the YLZ and HZ varieties were relatively high. Gallic acid and caffeic acid exert anti-colon cancer effects by inhibiting the COX-2/PGE2 pathway and eliminating ROS [39], and the polysaccharides in jujube fruit have been proven to have effects on tumours [40], blood lipids [41] and intestines [42]. The HMDZ variety is characterized by high contents of alkaloid substances. Daidzein and hesperidin induce cell cycle arrest in breast cancer cells by regulating the PI3K/Akt and MAPK signalling pathways, whereas epicatechin alleviates alcoholic liver injury through the Nrf2/ARE pathway [43,44]. Moreover, the TP53 (p53) target and the MAPK signalling pathway have dynamic coupling and bidirectional interactions in the regulation of the G2 phase checkpoint induced by DNA damage, jointly determining cell fate (repair, apoptosis, or senescence) [45]. These active ingredients show tentative predictive associations with health-related biological properties, providing preliminary hypotheses for further experimental verification.

## 5. Conclusions

In this study, the metabolites of five jujube fruit varieties (HZ, JZ, KSGEXZ, YLZ, HMDZ) were systematically compared and analyzed using UPLC–MS/MS, network pharmacology, and molecular docking methods. A total of 1583 metabolites were identified, covering 13 major categories. Among them, alkaloids, amino acids and their derivatives were the main components. Among the five kinds of jujube fruits, HMDZ was rich in alkaloids, which is conducive to the development of characteristic functional products. JZ and HZ were characterized by high contents of organic acids and sugars, respectively, endowing the fruit with sweet and sour flavours. KSGEXZ was composed mainly of metabolites such as flavonoids, steroids, tannins, lignans and coumarins. YLZ had a relatively high content of phenolic substances. Each type of jujube had unique and significantly abundant dominant metabolites. Based on the computational database analysis, we predicted 141 active components in these 5 jujube varieties that may be related to health-related biological processes. The results of molecular docking analysis revealed that catechin, naringenin, and coniferin had high binding energies with the core target TP53 and bound through hydrogen bonds and hydrophobic interactions. This study reveals the metabolic differences among jujube cultivars and provides a theoretical basis for the quality evaluation, breeding, and high-value utilization of jujube germplasm resources.

### Limitations

This study adopted a widely targeted metabolomics approach to characterize metabolite profiles in jujube fruits, combined with network pharmacology and molecular docking to screen core active components and key targets. All results from network analysis and molecular docking belong to in silico predictive analyses, without direct in vitro or in vivo functional verification.

## Data Availability

The original contributions presented in the study are included in the article/Supplementary Materials. Further inquiries can be directed to the corresponding author.

## Supplementary Materials

The following supporting information can be downloaded at: https://www.mdpi.com/article/10.3390/foods15081347/s1. Figure S1: Overlap of the QC sample TICs; Figure S2: Distributions in the CVs for the samples in each group. Different colours represent different grouped samples, and QC represents the quality control sample; Figure S3: OPLS-DA verification diagram. (a) HZ vs. HMDZ. (b) JZ vs. HMDZ. (c) JZ vs. HZ. (d) KSGEXZ vs. HMDZ. (e) KSGEXZ vs. HZ. (f) KSGEXZ vs. JZ. (g) YLZ vs. HMDZ. (h) YLZ vs. HZ. (i) YLZ vs. JZ. (j) YLZ vs. KSGEXZ; Figure S4: OPLS-DA pairwise comparisons of the differentially abundant metabolite scores. (a) HZ vs. HMDZ. (b) JZ vs. HMDZ. (c) JZ vs. HZ. (d) KSGEXZ vs. HMDZ. (e) KSGEXZ vs. HZ. (f) KSGEXZ vs. JZ. (g) YLZ vs. HMDZ. (h) YLZ vs. HZ. (i) YLZ vs. JZ. (j) YLZ vs. KSGEXZ; Figure S5: Volcano plots showing the contents of the differentially abundant metabolites. (a) HZ vs. HMDZ. (b) JZ vs. HMDZ. (c) JZ vs. HZ. (d) KSGEXZ vs. HMDZ. (e) KSGEXZ vs. HZ. (f) KSGEXZ vs. JZ. (g) YLZ vs. HMDZ. (h) YLZ vs. HZ. (i) YLZ vs. JZ. (j) YLZ vs. KSGEXZ; Figure S6: Enrichment map of the pairwise comparisons of the differentially abundant metabolites. (a) HZ vs. HMDZ. (b) JZ vs. HMDZ. (c) JZ vs. HZ. (d) KSGEXZ vs. HMDZ. (e) KSGEXZ vs. HZ. (f) KSGEXZ vs. JZ. (g) YLZ vs. HMDZ. (h) YLZ vs. HZ. (i) YLZ vs. JZ. (j) YLZ vs. KSGEXZ; Table S1: The relative contents of active substances identified in fruit of 5 jujube varieties; Table S2: Comparisons of the differentially abundant metabolites in the 5 jujube varieties; Table S3: Up regulation and down regulation of metabolites in the control group of five jujube varieties; Table S4: Common diseases possibly related to jujube; Table S5: The key active ingredient in jujube fruit has health function; Table S6:The topological structure characteristics of the main targets; Table S7: The binding energy between the core target TP53 and the key active substances in jujube fruits.
