# Supplementary material for: Integrated Metabolomics and Network Pharmacology Reveal the Active Components and Potential Health-Beneficial Mechanisms of Different Jujube (Ziziphus jujuba) Cultivars
Source: Foods. 2026 Apr 13;15(8):1347. doi: 10.3390/foods15081347 (PMC13115148; doi:10.3390/foods15081347)
Supplement: Supplementary file 1 [file foods-15-01347-s001.zip › Supplementary Figures.pdf]

Supplementary Figures

a

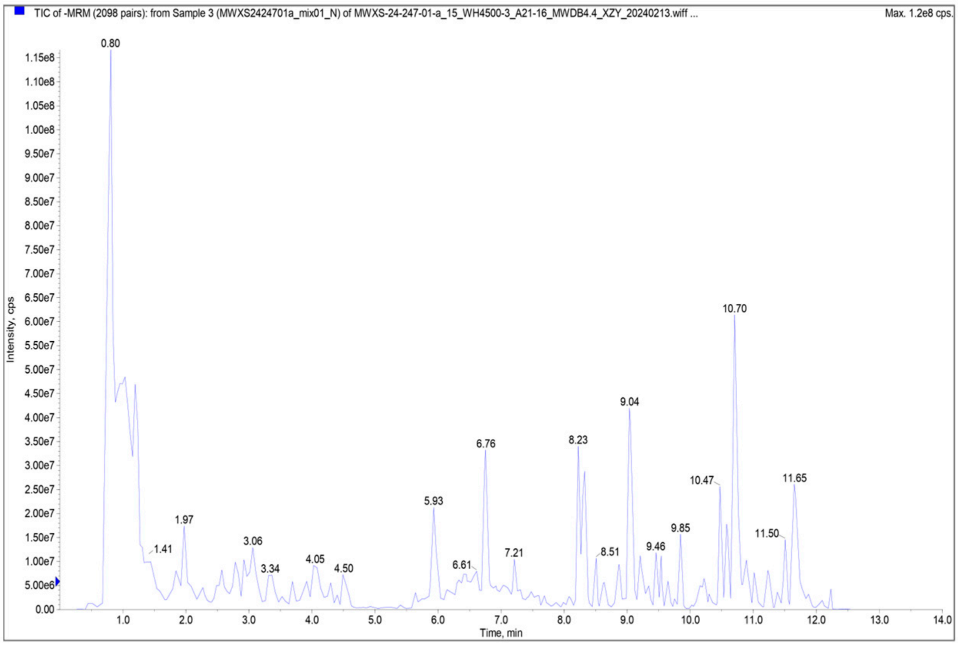

b

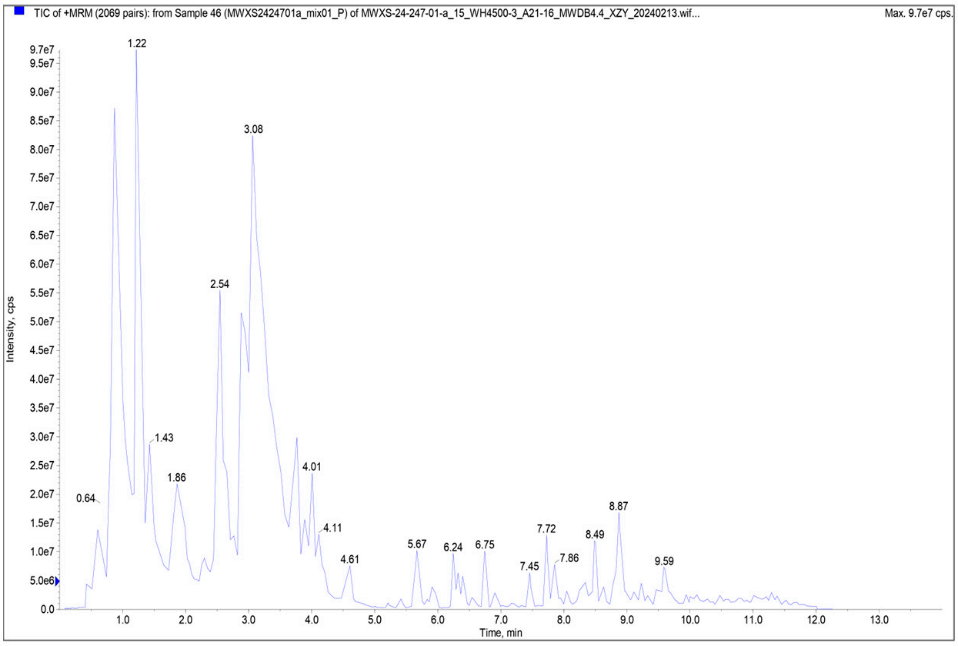

Notes: (a) indicates negative ion mode, (b) indicates positive ion mode.

Figure S1. Overlap of the QC sample TICs.

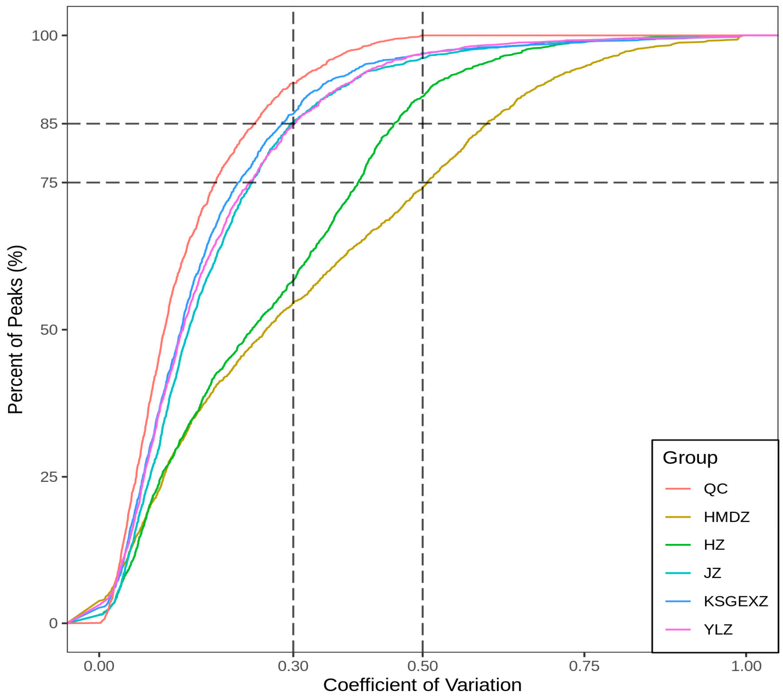

Figure S2. Distributions in the CVs for the samples in each group. Different colours represent different grouped samples, and QC represents the quality control sample.

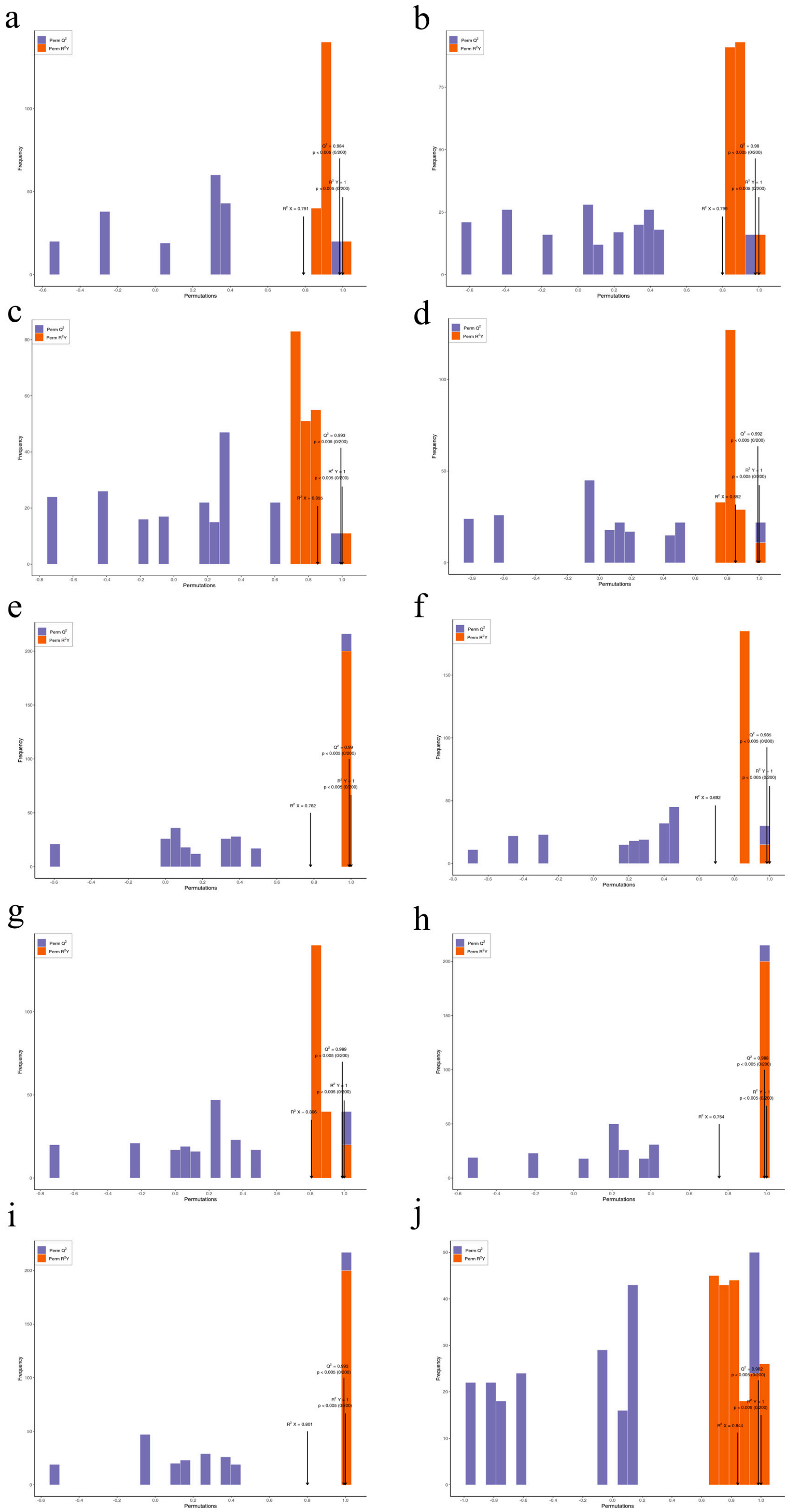

Figure S3. OPLS-DA verification diagram. (a) HZ vs HMDZ. (b) JZ vs HMDZ. (c) JZ vs HZ. (d) KSGEXZ vs HMDZ. (e) KSGEXZ vs HZ. (f) KSGEXZ vs JZ. (g) YLZ vs HMDZ. (h) YLZ vs HZ. (i) YLZ vs JZ. (j) YLZ vs KSGEXZ.

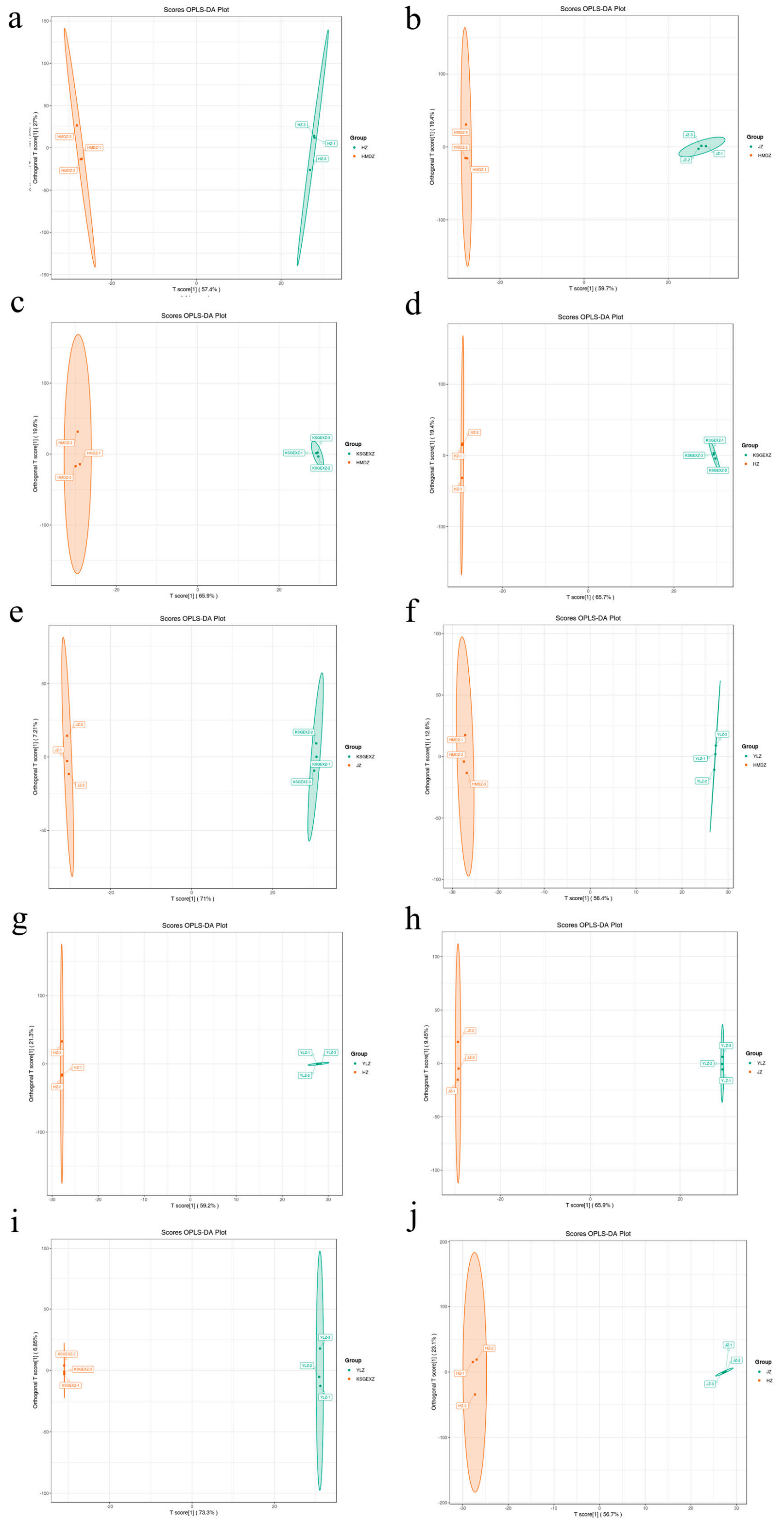

Figure S4. OPLS-DA pairwise comparisons of the differentially abundant metabolite scores. (a) HZ vs HMDZ. (b) JZ vs HMDZ. (c) JZ vs HZ. (d) KSGEXZ vs HMDZ. (e) KSGEXZ vs HZ. (f) KSGEXZ vs JZ. (g) YLZ vs HMDZ. (h) YLZ vs HZ. (i) YLZ vs JZ. (j) YLZ vs KSGEXZ.

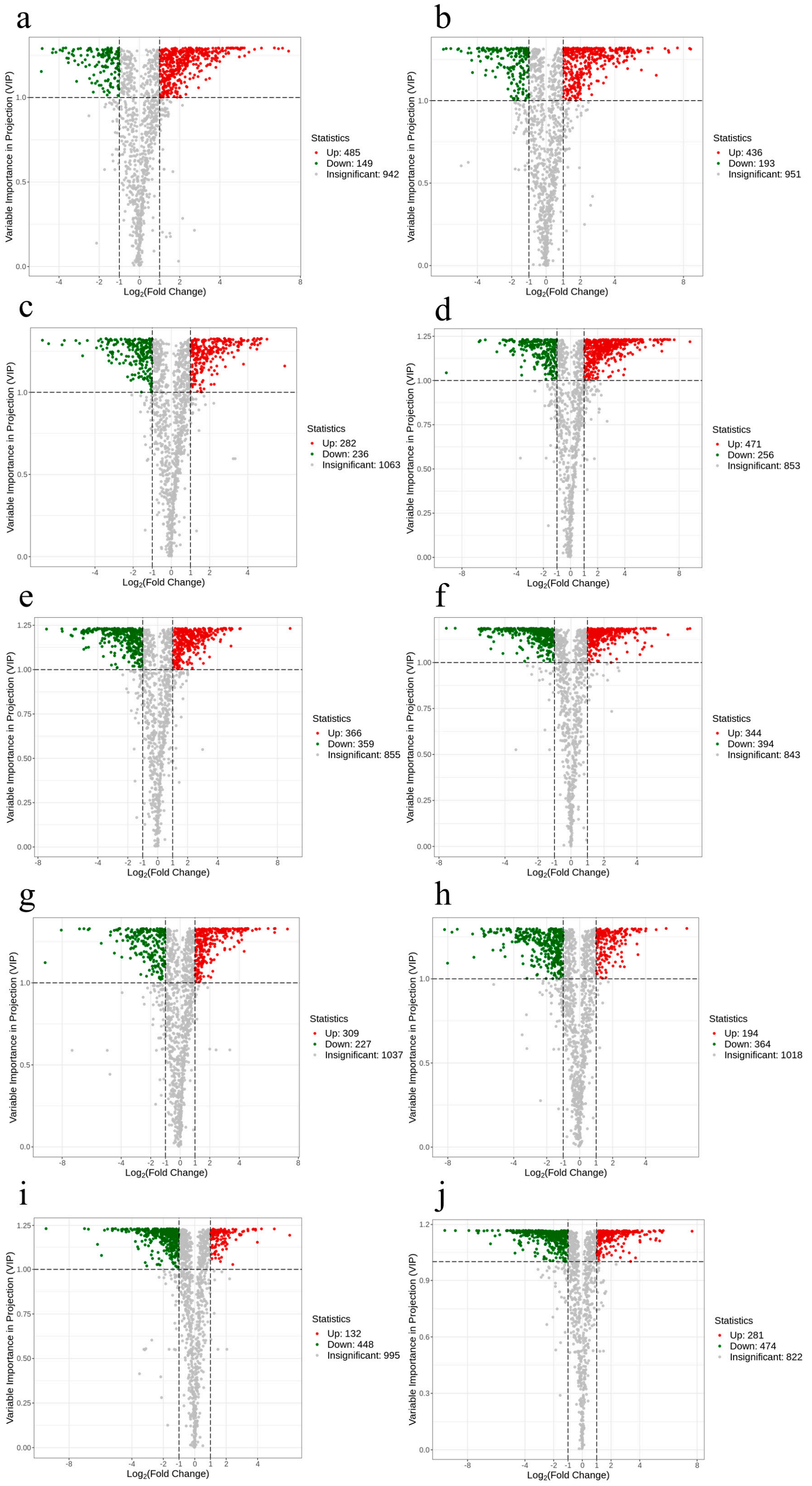

Figure S5. Volcano plots showing the contents of the differentially abundant metabolites. (a) HZ vs HMDZ. (b) JZ vs HMDZ. (c) JZ vs HZ. (d) KSGEXZ vs HMDZ. (e) KSGEXZ vs HZ. (f) KSGEXZ vs JZ. (g) YLZ vs HMDZ. (h) YLZ vs HZ. (i) YLZ vs JZ. (j) YLZ vs KSGEXZ.

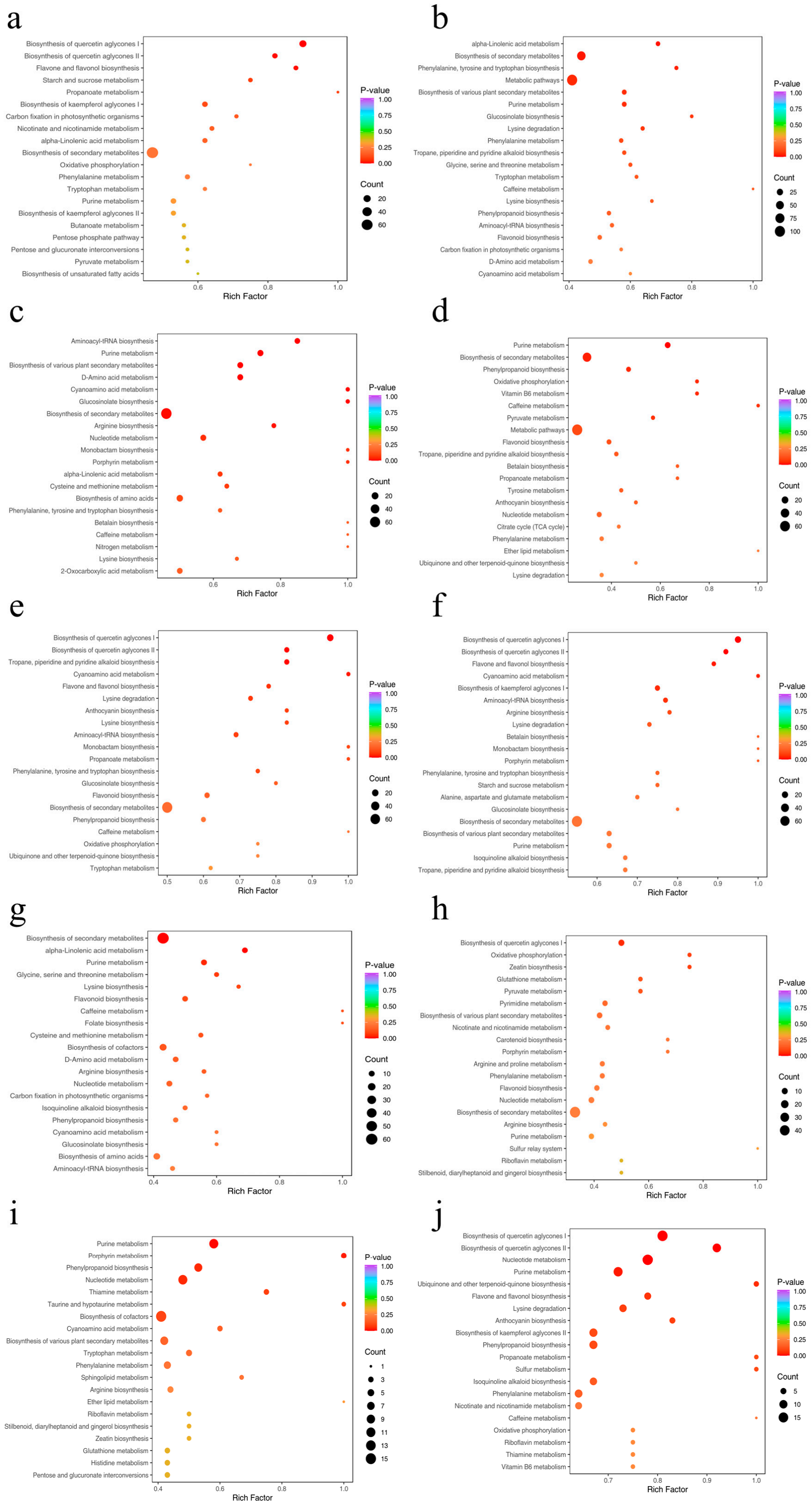

Figure S6. Enrichment map of the pairwise comparisons of the differentially abundant metabolites. (a) HZ vs HMDZ. (b) JZ vs HMDZ. (c) JZ vs HZ. (d) KSGEXZ vs HMDZ. (e) KSGEXZ vs HZ. (f) KSGEXZ vs JZ. (g) YLZ vs HMDZ. (h) YLZ vs HZ. (i) YLZ vs JZ. (j) YLZ vs KSGEXZ.
